# Antileishmanial Effect of 1,5- and 1,8-Substituted Fused Naphthyridines

**DOI:** 10.3390/molecules29010074

**Published:** 2023-12-22

**Authors:** Estela Melcón-Fernandez, Endika Martín-Encinas, Francisco Palacios, Gulio Galli, Rosa M. Reguera, María Martínez-Valladares, Rafael Balaña-Fouce, Concepción Alonso, Yolanda Pérez-Pertejo

**Affiliations:** 1 Departamento de Ciencias Biomédicas, Facultad de Veterinaria, Universidad de León, Campus de Vegazana s/n, 24071 León, Spainggal@unileon.es (G.G.);; 2 Departamento de Química Orgánica I, Facultad de Farmacia, Lascaray Research Center, Universidad del País Vasco/Euskal Herriko Unibertsitatea (UPV/EHU), Paseo de la Universidad 7, 01006 Vitoria-Gasteiz, Spain

**Keywords:** visceral leishmaniasis, fused 1,5-naphthyridines, fused 1,8-naphthyridines, intramacrophagic *Leishmania* parasites, mouse intestinal organoids

## Abstract

In the absence of a vaccine, there is a need to find new drugs for the treatment of neglected tropical diseases, such as leishmaniasis, that can overcome the many drawbacks of those currently used. These disadvantages include cost, the need to maintain a cold chain, the route of administration, the associated adverse effects and the generation of resistance. In this work we have evaluated the antileishmanial effect of 1,5- and 1,8-substituted fused naphthyridines through in vitro and ex vivo assays, using genetically modified axenic and intramacrophagic *Leishmania infantum* amastigotes. The toxicity of these compounds has been tested in the mammalian host cell using murine splenic macrophages, as well as in murine intestinal organoids (miniguts) in order to assess their potential for oral administration. The 1,8- derivatives showed greater leishmanicidal activity and the presence of a nitrogen atom in the fused ring to the naphthyridine was important to increase the activity of both types of molecules. The aromatization of the pyridine ring also had marked differences in the activity of the compounds.

## 1. Introduction

Current treatment of leishmaniasis, a complex of vector-borne diseases caused by parasitic protists of the genus *Leishmania*, remains a challenge [1,2,3]. The lack of a vaccine makes chemotherapy the only way to manage the infection when the first symptoms appear. However, the ability of the parasite to evade the host immune system and the intracellular localization of the parasite within host macrophages are pitfalls that hinder and delay the discovery and development of new drugs to treat these diseases [4]. Last but not least, leishmaniasis is classified by the WHO as a neglected tropical disease widely spread in the tropical and subtropical areas of the planet [5], where the drugs currently in use are scarce, outdated and expensive. In addition, current drugs exhibit toxicity, adverse side effects, poor oral bioavailability and loss of efficacy due to the increasing emergence of resistant strains [1,6,7].

Despite their significant poor tolerability, pentavalent antimonials are still the first-line treatment for different types of leishmaniasis. In addition to their cardiotoxicity, they require prolonged and painful parenteral administration, which makes adherence difficult [8]. Formulations of the polyene fungicide amphotericin B are strongly effective against the visceral form of the disease, but they must be administered by intravenous infusions and require a cold chain to reach the point of care [4,9]. Finally, miltefosine is the only approved oral antileishmanial drug, but unfortunately, it cannot be administered in pregnant women due to its teratogenicity and easily promotes resistance [9]. Combinations of these drugs and with the antibiotic paromomycin represent an interesting scenario until more effective and safer molecules are registered [10,11]. Given the limited number of drugs currently used against leishmaniasis and their drawbacks, it is urgently needed to search for new compounds that allow short, safe, inexpensive treatments and whose requirements are in line with the needs of developing countries (target product profile), where the disease is most prevalent [12].

There is a broad consensus among medicinal chemists that the presence of nitrogen atoms in the heterocyclic structure is relevant to improve interactions in biological systems as nitrogenated heterocycles are part of the chemical structure of many natural products and agents with significant biological activity, such as antiviral, antibiotic and antitumor drugs [13,14,15,16,17,18,19,20]. In fact, the FDA database reveals that a large number of accepted drugs contain a nitrogen heterocycle in their structure [21]. Therefore, in the drug discovery process, the development of practical synthetic routes to access these structural motifs in the simplest way possible is an important goal for synthetic and medicinal chemists. 

One of the most straightforward and versatile processes for the preparation of heterocyclic nitrogen compounds is the Povarov reaction [22]. With this in mind, our group has developed a suitable, efficient, fast and versatile methodology for the synthesis of nitrogenated heterocycles and good results were observed as topoisomerase IB (TopIB) inhibitors of these derivatives, with antileishmanial activity [23,24].

In addition, if the traditional Povarov reaction represents an excellent tool for the preparation of bicyclic nitrogenated heterocycles, such as the aforementioned quinoline or naphthyridine derivatives, compounds with a greater number of fused cycles can also be prepared with this methodology in its intramolecular version [25]. In these cases, the aza-Diels–Alder process (a [4+2] cycloaddition reaction) involves an aldehyde functionalized with a dienophile and an aromatic amine. Moreover, if the aromatic amine used is heterocyclic such as 2-aminopyridine or 3-aminopyridine, this process allows the direct preparation of fused heterocycles with several heteroatoms in their structure.

Under this context, the objective of the present study was to screen a series of fused 1,5- and 1,8-naphthyridines to evaluate their antileishmanial activity by using in vitro and ex vivo tests. The antiparasitic activity of these compounds was assessed in both axenic and intramacrophagic forms of *Leishmania infantum* using an in-house improved bioimaging system. In addition, the potential toxicity of the assayed compounds was evaluated in mouse splenic macrophages and murine intestinal organoids in order to assess their tolerability as oral drugs.

## 2. Results

### 2.1. Chemistry

**Scheme 1:**
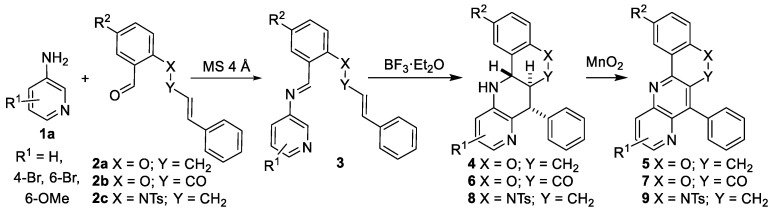
Intramolecular Povarov reaction of 3-aminopyridine **1a** with functionalized aldehydes **2**.

**Figure 1:**
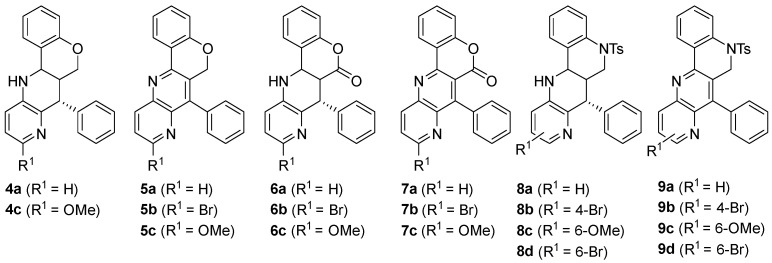
Structures of fused 1,5-naphthyridine derivatives **4**–**9** tested as antileishmanial agents.

**Scheme 2:**
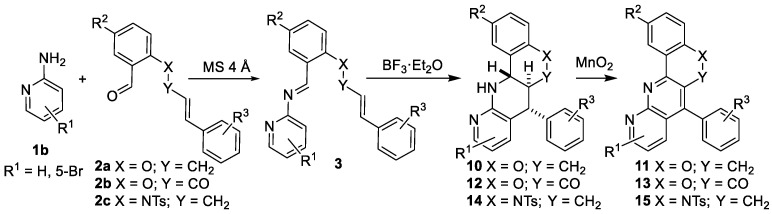
Intramolecular Povarov reaction of 2-aminopyridine **1b** and functionalized aldehydes **2**.

The synthesis of fused 1,5-naphthyridines and 1,8-naphthyridines is depicted in Scheme 1 and Scheme 2. The synthesis of fused 1,5-naphthyridines (Scheme 1) was performed by an intramolecular Povarov type [4+2] cycloaddition reaction. Thus, when 3-aminopyridine **1a** was reacted with functionalized aldehydes **2** in refluxing chloroform, in the presence of 2 equivalents of BF_3_·Et_2_O, tetrahydro[1,5]naphthyridine derivatives **4**, **6**, **8** (Scheme 1, Figure 1) were regio- and diastereoselectively obtained as previously reported by us [26,27].

**Figure 2:**
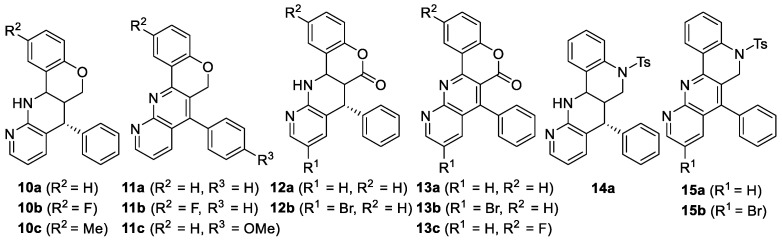
Structures of fused 1,8-naphthyridine derivatives **10**–**15** tested as antileishmanial agents.

When 2-aminopyridine **1b** was used tetrahydro[1,8]naphthyridines derivatives **10**, **12**, **14** (Scheme 2, Figure 2) were isolated as well in a regio- and diastereoselective way [28].

In addition, the subsequent dehydrogenation of tetrahydronaphthyridine derivatives with MnO_2_ in refluxing toluene yielded the corresponding 1,5-naphthyridines **5**, **7** and **9** (Scheme 1, Figure 1) and 1,8-naphthyridines **11**, **13** and **15** (Scheme 2, Figure 2), respectively, as previously reported [26,27,28]. The described methodologies represent an easy and efficient strategy for the preparation of these compounds containing a wide range of electron-releasing and electron-withdrawing substituents.

### 2.2. In Silico ADME

In silico ADME studies, also known as computer-based studies, [29,30,31,32] play a crucial role in the drug discovery and development process. ADME stands for absorption, distribution, metabolism and excretion, which are key factors that determine the pharmacokinetics and efficacy of a drug. In silico ADME studies involve the use of computational models and algorithms to predict the ADME properties of a drug which not only saves time and resources, but also helps in the identification of potential safety concerns.

Molecular properties such as the partition coefficient (Log P o/w), molecular weight, hydrogen bond donors and acceptors, topological polar surface area (TPSA) and violation of Lipinski’s rule of five were assessed (Table 1).

In general, a drug candidate with a LogP value between 0 and 5 is considered to have favorable ADME properties. A LogP value that is too low may indicate poor lipid solubility, which can affect the absorption and distribution of the drug in the body. A LogP value that is too high may indicate poor aqueous solubility, which can lead to poor bioavailability and potential toxicity due to the accumulation of the drug in fatty tissues. In the case of our compounds, they have a LogP value between 2.5 and 4 (with a mean of 3.12), so they would fall within the favorable range mentioned above.

TPSA, or the topological polar surface area, is another commonly used parameter in drug discovery and development to predict the ADME properties of a drug candidate. TPSA is a measure of the polar surface area of a compound, which is important for its interaction with biological targets and its ability to cross biological membranes. In general, a drug candidate with a TPSA value between 20 and 140 Å^2^ is considered to have favorable ADME properties. In Table 1, all the compounds tested in this work fall within the favorable range for good oral bioavailability.

Finally, Lipinski’s Rule of Five [33], a widely used rule in drug discovery to assess the drug-like properties of a compound, was performed to study our compounds. It is recommended that the orally active drug candidate should not have more than one violation of Lipinski’s rule. All compounds tested except compounds **8b**, **8d**, **9b**, **9d** and **15b** meet the established criteria (Table 1).

The absorption of a drug is a critical factor in determining its efficacy and safety. It refers to the process by which a drug enters the bloodstream and reaches its target site of action. Understanding the factors that influence drug absorption is essential for optimizing drug therapy and minimizing adverse effects. Because of this, the absorption of drug was evaluated based on aqueous solubility, intestinal absorption and permeability (Table 2). Thus, the aqueous solubility (Log S) of all compounds ranges from −7.48 to −4.40 log mol/L, which shows the moderate solubility in water of the synthesized compounds. In addition, all of them show intestinal absorption above 93%, which is interesting because most orally administered drugs are absorbed mainly through the small intestine due to its large surface area. Finally, the Caco-2 permeability, the logarithm of the apparent permeability coefficient (log Papp > 8 × 10^−6^ cm/s), was predicted. Since the compound is considered to have high Caco-2 permeability if the predicted value is >0.90, in our case, all the synthesized compounds have high Caco-2 permeability.

Finally, the volume of distribution (VDss), blood–brain barrier permeability (BBB permeability) [34,35] and the fraction of unbound of the synthesized compounds were further assessed. Considering that a Log VDss > 0.45 L/kg indicates a high volume of drug distribution, we can say that all synthesized compounds have low to moderate volume of distribution in tissues, except for compound **5a**. On the other hand, a compound is said to be readily permeable across the BBB if the predicted value of log BBB is >0.3 and poorly distributed if the value is <−1. In our estimations it can be seen that chromeno[4,3-*b*][1,5]naphthyridines (compounds **4**, **5**, **6** and **7**) and chromeno[4,3-*b*][1,8]naphthyridines (compounds **10**, **11**, **12** and **13**) show excellent BBB parameters but those fused to quinolines (compounds **8**, **9**, **14** and **15**) are not easily permeable through the BBB.

Additional factors to consider in drug development include metabolite formation mediated by cytochrome P450 (CYP) enzyme activities [36] and drug clearance (CL) [37]. CYP450 enzymes are a family of heme-containing enzymes that are involved in the metabolism of a wide range of endogenous compounds and xenobiotics. These enzymes play a crucial role in drug metabolism, and their activity can influence the efficacy and safety of many therapeutic agents. Compounds were studied as possible CYP2D6, CYP3A4, CYP1A2, CYP2C19 and CYP2C9 enzyme inhibitors. Noteworthy, all compounds show the ability to inhibit the CYP2C19 enzyme. Compounds with a methoxy group in their structure (**5c**, **9c** and **11c**) show inhibition of CYP2C9, which is primarily expressed in the liver and plays a critical role in the metabolism of numerous drugs. Conversely, the total clearance is primarily a combination of hepatic as well as renal clearance and is measured by the proportionality constant CLtot in log(mL/min/kg). The predicted value of all the synthesized compounds shows low CLtot ranging from −0.154 to 1.91. These results clearly indicate that all these compounds show appropriate pharmacokinetic properties and can be considered antileishmanial agents.

**Figure 3:**
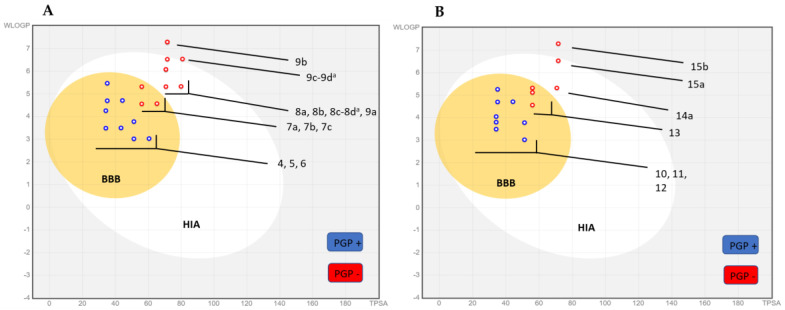
Boiled egg illustration of 1,5-naphthyridines (**A**) and 1,8-naphthyridines (**B**). The white region is the physicochemical space of molecules with the highest probability of being absorbed by the gastrointestinal tract, and the yellow region (yolk) is the physicochemical space of molecules with the highest probability of permeating into the brain. In the illustration, several compounds are represented with the same spot.

The boiled egg model [38] visually represents some of the ADME parameters of the evaluated 1,5- and 1,8-naphthyridines (Figure 3). Compounds that would cross both the blood–brain barrier (BBB) and human gastrointestinal tract (HIA) are placed in the yolk and those with only HIA-absorption in the white. Most of the tested compounds have positive BBB and HIA permeability, except compounds **8**, **9a**, **14a** and **15a** which only show positive HIA and compounds **9b**–**d** and **15b** which do not show results for either parameter.

P-glycoprotein (PGP) is a transporter protein found in the membrane of cells in various tissues, such as the gastrointestinal tract, liver and kidneys [39,40]. Its main function is to transport foreign substances, such as drugs, out of cells. Therefore, it may be desirable for a drug to be a PGP substrate, as it can help protect against toxicity or unwanted side effects. Dots colored blue correspond to molecules that are predicted to be P-glycoprotein substrates (PGP+) and are therefore actively pumped from the brain or into the gastrointestinal lumen (Figure 3). If they are predicted not to be P-glycoprotein substrates (PGP−), the corresponding dot appears in red. In the case of our tested compounds, it should be noted that quinolino naphthyridine derivatives **8**, **9**, **14** and **15** and chromeno naphthyridine derivatives **7** and **13** do not qualify as PGP-substrate candidates. In contrast, all the other compounds, derivatives **4**, **5**, **6**, **10**, **11** and **12**, are suitable as PGP substrate candidates.

### 2.3. Antileishmanial Effect

The 1,5- and 1,8-fused naphthyridine derivatives were evaluated as antileishmanial agents in assays using two forms of *L. infantum* amastigotes: axenic amastigotes recovered from bone marrow cells of infected BALB/c mice and intramacrophagic amastigotes obtained from splenic explants of the same subjects. The *L. infantum* strain used is a genetically modified strain previously engineered by our group to constitutively express the infrared protein iRFP from *Rhodopseudomonas palustris* bacteriophytochrome (iRFP *L. infantum*) [41,42]. This strain allows monitoring the viability of both forms of the parasite by quantifying the fluorescence emitted at 700 nm by the iRFP protein produced by living amastigotes [42].

Amastigotes obtained from mouse bone marrow cells were used to perform a first screening with the complete series of fused 1,5- and 1,8-naphthyridines. Molecules that did not inhibit amastigote growth at a concentration of 25 µM were considered to be poorly active and were discarded for subsequent assays on intramacrophagic amastigotes ex vivo. In the case of the tests carried out with intramacrophage amastigotes, the compounds that showed activity at a concentration lower than 20 µM were selected to perform dose–response curves, obtaining their corresponding EC_50_ values. The toxicity of these selected compounds was assessed in non-infected splenic cells and the cytotoxic concentration (CC_50_) value obtained was used to calculate the selective index (SI). The oral tolerability of these same compounds was tested by calculating the viability of murine intestinal organoids exposed to two concentrations (50 and 25 µM).

Table 3 shows the results obtained with the chromeno[4,3-*b*][1,5]naphthyridines and chromeno[4,3-*b*][1,5]naphthyridines-6-one derivatives. 

Of these compounds, only two were candidates to be tested in intramacrophagic amastigotes (**6b** and **7c**), obtaining EC_50_ values of 12.86 ± 0.82 µM and 36.99 ± 2.87 µM, respectively. However, the SI of both molecules was not very high (4.3 and 2.6), and the toxicity in murine intestinal organoids was similar to that observed in mouse splenic macrophages cells.

The replacement of the oxygen atom by nitrogen in these types of molecules produced more active compounds (Table 4). In this group, we found the more active and selective compounds such as molecules **8b** and **8c**, with EC_50_ values of 5.53 ± 0.26 µM and 4.93 ± 0.35 µM, respectively, and SI values of >18.1 and >20.2. In the case of compound **8c** the viability of murine intestinal organoids was of 100% at 50 µM. Compound **9b** has the peculiarity of being less toxic in murine intestinal organoids than in splenic cells, obtaining an SI of only 4.6 while the viability in organoids was of 100% at 50 µM. This result could be related to the negative HIA permeability (Figure 3) of this compound. Another point to highlight among the compounds of this group is the greater activity of compounds that lack the aromatic naphthyridine ring versus those that have the aromaticity.

Four chromeno[4,3-*b*][1,8]naphthyridines and chromeno[4,3-*b*][1,8]naphthyridine-6-one derivatives were tested in intramacrophagic amastigotes (**10b**, **11a**, **13a** and **13b**, Table 5). Of these four, the highest SI was obtained with **11a** (EC_50_ = 7.70 ± 0.29 µM; SI ≥ 13). Compound **10b** was less toxic in intestinal organoids than in mouse splenic macrophages, showing 90.04% of viability in organoids at 50 µM against of a CC_50_ value of 50.72 ± 0.99 µM in mouse splenic macrophages. The in silico predictions for this compound have not identified problems relative to its HIA absorption. Contrary to 1,5-naphthyridines (Table 4) compounds in this group were more active with the aromatic naphthyridine ring.

The three available 1,8-derivatives with nitrogen atoms instead of oxygen were tested in intramacrophagic amastigotes (Table 6). However, their SI was low and the toxicity in organoids was similar than obtained with splenic cells.

## 3. Discussion

Naphthyridine derivatives have been associated with many biological activities, and the 1,5- and 1,8-naphthyridine scaffolds have been used in the generation of drugs with antileishmanial, antimicrobial, antiviral, anthelmintic or antimalarial activities [23,24,43,44,45,46]. Looking for new drugs to overcome the drawbacks of those currently used and that have been previously mentioned, we have evaluated the antileishmanial activity of substituted fused 1,5- or 1,8-naphthyridines, which has enabled a structure–activity relationship (SAR) study. SAR analysis is a fundamental approach that investigates the links between the structure of chemical compounds and their observed biological or physicochemical properties. By systematically studying the relationships between structural features and activity, SAR analysis provides valuable insights for rational compound design and optimization.

**Figure 4:**
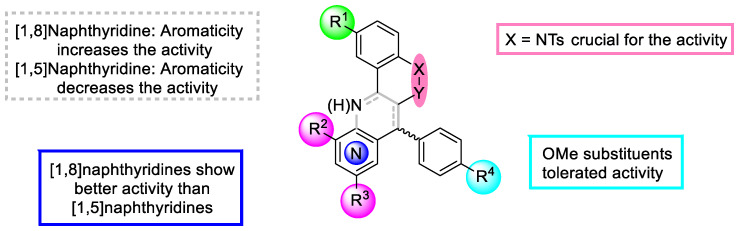
Structure–activity relationship analysis.

Among the six different isomeric forms of naphthyridines described, based on the position of the nitrogen atoms in the bicyclic system, the 1,8-naphtyridines have been one of the most studied. Based on the obtained biological results and observing the structural differences of the tested compounds, it has been observed that 1,8-naphthyridine derivatives exhibit better cytotoxic activity than 1,5-naphthyridines. Furthermore, it is worth noting that in 1,5-naphthyridines, the aromatization of the pyridine ring decreases biological activity, while in the 1,8-naphthyridines, an increase is observed. Besides, the presence of a nitrogen atom (X = NTs) in the fused ring to the naphthyridine is crucial for increase of the biological activity (Figure 4).

Previous studies with 1,8-fused naphthyridines, focused on the mechanism of action of these compounds, showed that only five compounds (**10a**, **13a**, **14a**, **15a**, **15b**) had an inhibitory effect on DNA relaxation activity using a recombinant human DNA topoisomerase IB (hTopIB) at a 100 mM final concentration. However, these compounds had cytotoxic effects on two cancer cell lines at much lower concentrations (1–15 μM range), so it could be concluded that the inhibitory effect on hTopIB did not correspond to its cytotoxicity. With the exception of compound **10a**, which was not effective against *Leishmania* amastigotes, the three tosylated derivatives (**14a**, **15a** and **15b**) and the 1,8-chromene derivative fused to naphthyridine (**13a**) showed significant antileishmanial activity, but also significant toxicity. These compounds, however, did not show leishmanial DNA topoisomerase IB (LdTopIB) inhibitory activity (Supplementary Materials), thus concluding the marginal relevance of this target in the mechanism of action of these compounds [26,27,28].

Some 1,8-naphthyridines have been investigated as antitubercular drugs and their activity has been explained by the inhibition of the bacterial DNA gyrase a type II topoisomerase [47]. The antiproliferative activity of some 1,8-naphthyridines like voreloxin is believed that to be related to topoisomerase II inhibition [48]. Other 1,8-naphthyridines have been designed as topoisomerase II inhibitors, studying the intercalation with DNA and enzymes [49,50]. Given that none of the compounds tested in this work were inhibitors of topoisomerase IB, based on the existing literature they could be acting as inhibitors of DNA topoisomerase II.

In conclusion, in the more aromatized 1,5-naphthyridines, the activity decreases (compare **7c** with **6b**), whereas in the more aromatized 1,8-naphthyridine derivatives (compare **12** and **13**), an increase in antileishmanial activity is observed. Furthermore, according to the biological results, 1,8-naphthyridines show better antileishmanial activity than 1,5-naphthyridine derivatives.

Outstanding in this study is the behavior of the quinoline derivatives. Better antileishmanial activity is observed for naphthyridines condensed with quinoline (compounds **8**, **9**, **14**, **15**) than when condensed with a chromene ring (compounds **4**, **5**, **10** and **11**) or chromenone (compounds **6**, **7**, **12**, **13**). Therefore, the substitution of the oxygen atom for the NTs group in the structure of these heterocyclic derivatives results in a very significant increase in antileishmanial activity. While chromeno[4,3-*b*][1,5]naphthyridine **6b** stands out for an EC_50_ value of 12.86 ± 0.82 µM, quinoline derivatives **8a**–**c** show EC_50_ values between 4.93–5.53 µM and are less toxic, with higher selectivity indices. Intracellular inhibition of amastigotes is observed for quinolino[4,3-*b*][1,8]naphthyridine derivatives, while this does not occur for 1,8-naphthyridines condensed with chromene or chromenone.

These findings provide valuable insights into the structure–activity relationship of the compounds studied, allowing for a deeper understanding of the factors influencing their biological activity. The results contribute to the rational design and optimization of future compounds with improved bioactive properties.

## 4. Materials and Methods

### 4.1. Drugs

The standard antileishmanial drug amphotericin B (AMB) (Fisher Scientific, Waltham, MA, USA) was dissolved in DMSO to a final concentration of 50 mM. The 1,5- and 1,8- fused naphthyridines (>99% purity) from Figure 1 and Figure 2 were dissolved in DMSO (Sigma-Aldrich, Merck, Darmstadt, Germany) to a final concentration of 100, 50 or 25 mM depending on their solubility.

### 4.2. Experimental Animals and Ethical Statement

Experimental infections to obtain primary axenic bone marrow amastigotes or splenic explants harboring *L. infantum* amastigotes (intramacrophagic amastigotes) were carried out using female Balb/c. Animals were purchased from Janvier Laboratories (St Berthevin Cedex, France) and were maintained in the animal house at the University of León under standard housing conditions, with free access to food and water. The animal handling protocols used in this study complies with Spanish Act (RD 53/2013) inspired by European Union Legislation (2010/63/UE) and were approved by the Junta de Castilla y León under the authorization OEBA 007-2019.

### 4.3. Parasites

The Leishmania strain used for in vitro and ex vivo studies was derived from *L. infantum* BCN, which was genetically modified in house to constitutively produce the infrared fluorescent protein (iRFP): iRFP-*L. infantum* for detection of viable parasites [41,42]. Promastigotes were routinely grown in Schneider’s insect medium (Sigma-Aldrich, Merck, Darmstadt, Germany) supplemented with 20% (*v*/*v*) fetal calf serum (FBS) and antibiotic cocktail (100 U/mL penicillin and 100 µg/mL streptomycin) at 26 °C until infections in mice.

### 4.4. Experimental Infections and Set Up of Primary Cultures

Balb/c mice (six to eight weeks old) were inoculated intraperitoneally with 1.5 × 10^9^ iRFP-*L. infantum* metacyclic promastigotes. After 8 to 10 weeks post inoculation, mice were humanely sacrificed, aseptically dissecting spleen, as well as femur and tibia of both paws.

To obtain iRFP-*L. infantum* axenic amastigotes, femur and tibia were carefully cut at both ends, and marrow cells were removed by passing PBS through the medullary cavity with a 29G needle [51]. The bone marrow cell suspension was filtered through a 100 µm cell strainer and centrifuged at 3500× *g* for 10 min at room temperature. After centrifugation, cells were resuspended in amastigote culture medium: 15 mM KCl; 136 mM KH_2_PO_4_; 10 mM K_2_HPO_4_·3H_2_O; 0.5 mM MgSO_4_. 7H_2_O; 24 mM NaHCO_3_; 22 mM glucose; 1 mM glutamine, 1xRPMI 1640 vitamin mix (Sigma-Aldrich, Merck, Darmstadt, Germany), 10 mM folic acid, 100 mM adenosine, 1xRPMI amino acid mix (Sigma-Aldrich, Merck, Darmstadt, Germany), 5 mg/mL hemin, antibiotic cocktail, 25 mM MES and 10% FBS, and incubated at 37 °C.

Intramacrophagic amastigotes were obtained by cutting spleens into small pieces that were incubated for 25 min with 0.5 mL of collagenase D (2 mg/mL) (Merck, Darmstadt, Germany) dissolved in 4.5 mL buffer (10 mM HEPES, pH 7.4, 150 mM NaCl, 5 mM KCl, 1 mM MgCl_2_ and 1.8 mM CaCl_2_). The cell suspension was filtered through a 100 µm cell strainer. After erythrocyte lysis (150 mM NH_4_Cl, 1 mM KHCO_3_, 0.1 mM EDTA), splenocytes were centrifuged three times at 500× *g* for 7 min at 4 °C, with PBS washes between each centrifugation. The splenocyte suspension containing intramacrophagic amastigotes was resuspended in RPMI medium (Gibco, Fisher Scientific, Waltham, MA, USA) supplemented with 20% FBS, 1 mM sodium pyruvate, 24 mM NaHCO_3_, 2 mM L-glutamine, 1× RPMI vitamins, 25 mM HEPES and antibiotic cocktail.

### 4.5. Axenic and Intramacrophagic Amastigotes Viability Assays

To test the antileishmanial effect of naphthyridines on axenic amastigotes, 40 μL of a suspension containing 3 × 10^4^ iRFP-*L. infantum* parasites per well were incubated with another 40 μL of the drugs dilutions in amastigote culture medium with 7.5% FBS. Incubations were performed in 384-well black microtiter plates with an optical bottom at 37 °C and 5% CO_2_ for 72 h. Solutions of 0.1% (*v*/*v*) DMSO and 10 μM AMB were used as negative and positive controls, respectively. The assays were performed by triplicate.

For the ex vivo intramacrophagic amastigote assay, 40 μL of one-half or one-third serial dilutions of each diluted in supplemented RPMI medium, were added to black 384-well optical-bottom black plates containing 40 μL of murine splenocytes obtained as described above. Positive controls (containing 10 μM of AMB) and negative controls (containing 0.1% *v*/*v* DMSO) were included. The plates were incubated at 37 °C and 5% CO_2_, for a maximum period of 72 h and the assays were performed by triplicate.

The viability of axenic and intramacrophage amastigotes was calculated measuring the fluorescence emitted at 700 nm by the iRFP protein from live parasites and recorded by an Odyssey infrared imaging system (Li-Cor, NE, USA). The fluorescence emitted by the negative control wells was considered 100% viability, whereas the fluorescence emitted by the positive control wells, was adjusted to 0% viability.

Fluorescence emitted by intramacrophage amastigotes were plotted against the drug concentration using the nonlinear fit analysis provided by the Sigma Plot 10.1 statistical package to obtain EC_50_ values.

### 4.6. Cytotoxicity in Murine Splenic Cells and Selectivity Index Determination

In order to assess the in vitro safety of the naphthyridines, cytotoxicity assays were carried out in ex vivo splenic explants from uninfected mice, which were prepared as previously described to obtain intramacrophagic amastigotes. Cells were seeded at a density of 1 × 10^6^ cells per well in 96-well plates containing the prediluted compounds and incubated at 37 °C and 5% CO_2_ during 72 h. AlamarBlue assay (Invitrogen, Fisher Scientific, Waltham, MA, USA) was used to estimate cell viability following manufacturer’s recommendations. The assays were performed by triplicate.

Absorbance of viable cells vs. compound concentrations were fitted by nonlinear analysis using the Sigma Plot 10.1 statistical package to calculate the cytotoxic concentration 50 (CC_50_) values. Selectivity index (SI) was calculated as the ratio between CC_50_ and the EC_50_ values of intramacrophagic amastigotes.

### 4.7. Preparation of Murine Intestinal Organoids

Preparation and maintenance of murine intestinal organoids cultures were performed following the protocols published by Stemcell Technologies™ (https://www.stemcell.com/intestinal-epithelial-organoid-culture-with-intesticult-organoid-growth-medium-mouse-lp.html#protocols, accessed on 14 December 2023) with some modifications. Briefly, a section of small intestine from a Balb/c mouse was extracted and washed with ice-cold PBS. This section was cut into pieces around 2 mm each, washed 15–20 times with ice-cold PBS and incubated with Gentle Cell Dissociation Reagent (Stemcell Technologies™, Vancouver, BC, Canada) for 20 min. Then, the pieces were resuspended in ice-cold PBS with 0.1% BSA and left to settle at the bottom of the tube. The supernatant was collected and filtered through a 70 µm strainer. This process was repeated a total of four times obtaining different fractions. Cells from these fractions were centrifugated at 300× *g* for 5 min at 4 °C, washed with ice-cold PBS + 0.1% BSA and resuspended on 10 mL of ice-cold DMEM/F12 supplemented with glutamine and HEPES 15 mM (Gibco, Thermo Fisher Scientific, Waltham, MA, USA). After the evaluation under a microscope, the fraction with the highest crypt number but the lowest detritus presence was chosen. This fraction was centrifugated at 200× *g* for 5 min at 4 °C and the pellet was resuspended in a 1:1 mix of Geltrex GFR LDEV-free (Gibco, Fisher Scientific, Waltham, MA, USA) and IntestiCult™ Organoid Grow Medium Mouse (Stemcell Technologies™, Vancouver, BC, Canada). Fifty microliters of the suspension were pipetted into the middle of each well of a pre-heated 24-well plate and incubated at 37 °C for 10 min to allow polymerization of the matrix. In the end, 500 µL of IntestiCult™ Organoid Grow Medium was added to each well. The plates were incubated at 37 °C and 5% CO_2_, changing the culture medium every 48 h. Organoids were split 1 into 4 plates after 7 days.

### 4.8. Drug Tolerance in Murine Intestinal Organoids

Drug tolerance assays using murine intestinal organoids were performed in 384-well plates, following a protocol based on previous works and introducing several modifications [52]. Briefly, organoids were prepared discarding the culture medium and adding 800 µL of Gentle Cell Dissociation Reagent (Stemcell Technologies™, Vancouver, BC, Canada) directly on top of matrix domes. After 1 min incubation at room temperature, the matrix was broken by pipetting, and the content of the wells was retrieved. The suspension was incubated at room temperature for 10 min on a rocking platform at 20 rpm, and centrifugated at 300× *g* for 5 min at 4 °C. The pellet was resuspended in 10 mL of ice-cold DMEM/F12 (Gibco, Fisher Scientific, Waltham, MA, USA) supplemented with glutamine and HEPES 15 mM and centrifugated again at 200× *g* for 5 min at 4 °C. Cells were resuspended in a 1:1 mix of Geltrex GFR LDEV-free and IntestiCult™ Organoid Grow Medium Mouse (Stemcell Technologies™, Vancouver, Canada). The final volume of the mix was calculated to achieve a 4-fold dilution in the final plate compared to the original plate. The mix was distributed in a 384-well plate (8 µL/well) which was incubated at 37 °C for 10 min. After that, 32 µL of IntestiCult™ Organoid Grow Medium Mouse (Stemcell Technologies™, Vancouver, Canada) were added to each well. The plate was incubated at 37 °C and 5% CO_2_ and after 4 days, mature organoids were exposed to drugs, alone or combined. For positive control, hydrogen peroxide at the final concentration of 0.15 % *v*/*v* was used. DMSO at 0.2% was the negative control. Viability of organoids after 72 h of exposition was determined by the alamarBlue assay (Invitrogen, Thermo Scientific, USA).

### 4.9. Computational Assays—ADME Properties

The physicochemical and pharmacokinetics properties of tested compounds were calculated using swissADME [53] and pkCSM [54] online web servers. Chemical structures were imported in swiss ADME and pkCSM tools to calculate molecular as well as ADME properties of the compounds.

## Figures and Tables

**Table 1:** Physicochemical properties and drug-likeness for chromeno[4,3-*b*][1,5]naphthyridine and chromeno[4,3-*b*][1,8]naphthyridine derivatives. #-number.

Compound	LogP	Mol. Wt.	H-Donor	H-Acceptor	TPSA	Lipinski #Violations
**4a**	2.71	314.38	1	2	34.15	0
**4c**	2.76	344.41	1	3	43.38	0
**6a**	2.51	328.36	1	3	51.22	0
**6b**	2.69	407.26	1	3	51.22	0
**6c**	3.02	358.39	1	4	60.45	0
**8a**	3.11	467.58	1	3	70.68	0
**8b**	3.73	546.48	1	3	70.68	2
**8c**	3.81	497.61	1	4	79.91	0
**8d**	3.81	546.48	1	3	70.68	2
**5a**	3.26	310.35	0	3	35.01	0
**5b**	3.60	389.24	0	3	35.01	0
**5c**	3.60	340.37	0	4	44.24	0
**7a**	2.80	324.33	0	4	55.99	0
**7b**	3.12	403.23	0	4	55.99	0
**7c**	3.19	354.36	0	5	65.22	0
**9a**	3.70	463.55	0	4	71.54	0
**9b**	3.87	542.45	0	4	71.54	2
**9c**	3.97	493.58	0	5	80.77	0
**9d**	3.52	542.45	0	4	71.54	2
**10a**	3.05	314.38	1	2	34.15	0
**10b**	2.76	332.37	1	3	34.15	0
**10c**	2.92	328.41	1	2	34.15	0
**12a**	2.56	328.36	1	3	51.22	0
**12b**	2.94	407.26	1	3	51.22	0
**14a**	3.38	467.58	1	3	70.68	0
**11a**	2.99	310.35	0	3	35.01	0
**11b**	3.08	328.34	0	4	35.01	0
**11c**	3.27	340.37	0	4	44.24	0
**13a**	2.61	324.33	0	4	55.99	0
**13b**	3.00	403.23	0	4	55.99	1
**13c**	2.69	342.32	0	5	55.99	0
**15a**	3.48	463.55	0	4	71.54	0
**15b**	3.70	542.45	0	4	71.54	2

**Table 2:** Results of the ADME predictive study for chromeno[4,3-*b*][1,5]naphthyridine and chromeno[4,3-*b*][1,8]naphthyridine derivatives.

Compound	Log S (Log mol/L)	Caco-2 Perm. (Log Paap in 10^−6^ cm/s)	Int. Abs. (% abs)	VDss (L/kg)	Fract. Unb. (Fu.)	BBB Permeability (log BB)	CYP1A2 Inhibitor	CYP2C19 Inhibitor	CYP2C9 Inhibitor	CYP2D6 Inhibitor	CYP3A4 Inhibitor	Total Clearence Numeric (Log mL/min/kg)
**4a**	−4.73	1.779	96.038	0.312	0.027	Yes	No	Yes	No	Yes	Yes	0.180
**4c**	−5.00	1.195	94.748	−0.121	0.109	Yes	No	Yes	No	Yes	Yes	0.493
**6a**	−4.47	1.268	98,408	0.190	0.003	Yes	No	Yes	No	Yes	No	0.003
**6b**	−5.59	1.210	93.336	−0.277	0.122	Yes	No	Yes	No	No	No	−0.024
**6c**	−4.75	1.182	95.066	−0.303	0.112	Yes	No	Yes	No	Yes	Yes	0.379
**8a**	−6.21	1.153	98.895	−0.719	0.081	No	No	Yes	No	Yes	Yes	0.286
**8b**	−7.32	1.136	97.026	−0.669	0.073	No	No	Yes	No	No	No	−0.154
**8c**	−6.49	1.063	95.572	−0.961	0.238	No	No	Yes	No	Yes	Yes	0.392
**8d**	−7.12	1.140	97.414	−0.810	0.087	No	No	Yes	No	Yes	No	−0.103
**5a**	−4.92	1.660	100	0.525	0.26	Yes	Yes	Yes	No	Yes	Yes	0.834
**5b**	−6.03	1.143	96.994	0.099	0.354	Yes	Yes	Yes	No	No	Yes	0.135
**5c**	−5.18	1.122	98.725	0.025	0.343	Yes	Yes	Yes	Yes	Yes	Yes	0.881
**7a**	−5.00	1.410	100	0.403	0.368	Yes	Yes	Yes	No	No	No	0.921
**7b**	−6.11	1.137	98.66	0.193	0.363	Yes	Yes	Yes	No	No	No	0.363
**7c**	−5.27	1.098	100	0.108	0.352	Yes	Yes	Yes	Yes	No	Yes	1.091
**9a**	−6.36	1.119	100	−0.312	0.334	No	Yes	Yes	No	No	Yes	0.819
**9b**	−7.48	1.114	100	−0.293	0.333	No	Yes	Yes	No	No	No	0.137
**9c**	−6.64	1.167	99.946	−0.574	0.393	No	No	Yes	Yes	No	Yes	0.949
**9d**	−7.27	1.106	100	−0.386	0.338	No	Yes	Yes	No	No	No	0.123
**10a**	−4.92	1.765	96.706	0.142	0.035	Yes	No	Yes	No	Yes	No	0.577
**10b**	−5.07	1.803	94.633	−0.151	0.031	Yes	No	Yes	No	Yes	No	0.268
**10c**	−5.21	1.054	95.189	0.014	0.027	Yes	No	Yes	No	Yes	No	0.601
**12a**	−4.66	1.247	97.879	−0.184	0	Yes	No	Yes	No	Yes	No	0.467
**12b**	−5.56	1.044	94.333	−0.230	0.007	Yes	No	Yes	No	No	No	−0.092
**14a**	−6.40	0.431	98.124	−0.935	0.086	No	No	Yes	No	Yes	Yes	0.384
**11a**	−5.07	1.550	100	0.297	0.285	Yes	Yes	Yes	No	Yes	Yes	0.928
**11b**	−5.23	1.659	98.699	−0.046	0.306	Yes	Yes	Yes	No	No	Yes	0.777
**11c**	−5.13	1.121	100	0.131	0.294	Yes	Yes	Yes	Yes	Yes	Yes	0.905
**13a**	−5.16	1.393	100	0.273	0.344	Yes	Yes	Yes	No	No	No	1.068
**13b**	−6.06	1.101	99.395	0.046	0.349	Yes	Yes	Yes	No	No	No	0.046
**13c**	−5.31	1.430	100	0.028	0.346	Yes	Yes	Yes	No	No	No	1.006
**15a**	−6.51	1.000	100	−0.464	0.335	No	Yes	Yes	No	No	Yes	0.851
**15b**	−7.43	0.974	100	−0.596	0.341	No	No	Yes	No	No	No	−0.026

**Table 3:** Biological activity of chromeno[4,3-*b*][1,5]naphthyridines and chromeno[4,3-*b*][1,5]naphthyridine-6-ones. The antileishmanial effect was evaluated in axenic IRFP-*L.infantum* amastigotes from bone marrow cells and subsequently in intramacrophagic IRFP-*L.infantum* from murine spleens. Cytotoxicity was assessed in uninfected splenic macrophages and intestinal tolerance in murine gut organoids.

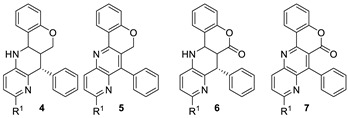							
Compound	R^1^	Axenic Amastigotes ^a^	Intracellular Amastigotes EC_50_ (µM)	Mouse Splenic Macrophages CC_50_ (µM)	SI	Intestinal Organoids	
						50 µM (%Viability)	25 µM (%Viability)
**4a**	H	>25	n.d.*	n.d	-		
**4c**	OMe	>25	n.d.	n.d.	-		
**5a**	H	>25	n.d.	n.d.	-		
**5b**	Br	>25	n.d.	n.d.	-		
**5c**	OMe	>25	n.d.	n.d.	-		
**6a**	H	>25	n.d.	n.d.	-		
**6b**	Br	<25	12.86 ± 0.82	55.66 ± 6.42	4.3	60 ± 7	72 ± 5
**6c**	OMe	>25	n.d.	n.d.	-		
**7a**	H	>25	n.d.	n.d.	-		
**7b**	Br	>25	n.d.	n.d.	-		
**7c**	OMe	<25	36.99 ± 2.87	97.68 ± 1.77	2.6	85 ± 13	99 ± 4

* n.d. not determined. ^a^ Compounds with inhibition at concentrations higher than 25 µM were considered not very active and were discarded for subsequent assays.

**Table 4:** Biological activity of quinolino[4,3-*b*][1,5]naphthyridines. The antileishmanial effect was evaluated in axenic IRFP-*L.infantum* amastigotes from bone marrow cells and subsequently in intramacrophagic IRFP-*L. infantum* from murine spleens. Cytotoxicity was assessed in uninfected splenic macrophages and intestinal tolerance in murine gut organoids.

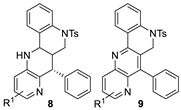							
Compound	R^1^	Axenic Amastigotes ^a^	Intracelullar Amastigotes EC_50_ (µM)	Mouse Splenic Macrophages CC_50_ (µM) *	SI	Intestinal Organoids	
						50 µM (%Viability)	25 µM (%Viability)
**8a**	H	<25	5.30 ± 0.71	45.03 ± 3.91	8.5	89 ± 10	93 ± 7
**8b**	4-Br	<25	5.53 ± 0.26	>100	>18.1	98 ± 13	>100
**8c**	6-OMe	<25	4.93 ± 0.35	>100	>20.28	>100	>100
**8d**	6-Br	<25	34.36 ± 9.70	>50	1.5	3 ± 1	4 ± 1
**9a**	H	>25	n.d.	n.d.	-		
**9b**	4-Br	<25	3.63 ± 0.15	16.89 ± 0.86	4.6	>100	>100
**9c**	6-OMe	>25	>20	n.d.	-		
**9d**	6-Br	>25	>20	n.d.	-		

* n.d. not determined. ^a^ Compounds with inhibition at concentrations higher than 25 µM were considered not very active and were discarded for subsequent assays.

**Table 5:** Biological activity of chromeno[4,3-*b*][1,8]naphthyridines and chromeno[4,3-*b*][1,8]naphthyridine-6-ones. The antileishmanial effect was evaluated in axenic IRFP-*L.infantum* amastigotes from bone marrow cells and subsequently in intramacrophagic IRFP-*L.infantum* from murine spleens. Cytotoxicity was assessed in uninfected splenic macrophages and intestinal tolerance in murine gut organoids.

	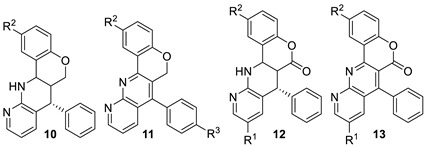								
Compound	R^1^	R^2^	R^3^	Axenic Amastigotes ^a^	Intracelullar Amastigotes EC_50_ (µM)	Mouse Splenic Macrophages CC_50_ (µM) *	SI	Intestinal Organoids	
								50 µM (%Viability)	25 µM (%Viability)
**10a**	-	H	H	>25	>20	n.d.	-		
**10b**	-	F	H	<25	7.57 ± 0.44	50.72 ± 0.99	6.7	90 ± 22	99 ± 8
**10c**	-	Me	H	>25	>20	n.d.	-		
**11a**	-	H	H	<25	7.70 ± 0.29	>100	>13	96 ± 1	92 ± 1
**11b**	-	F	H	>25	>20	n.d.	-		
**11c**	-	H	OMe	>25	>20	n.d.	-		
**12a**	H	H	-	>25	>20	n.d.	-		
**12b**	Br	H	-	>25	>20	n.d.	-		
**13a**	H	H	-	<25	10.58 ± 1.21	34.65 ± 1.79	3.3	53 ± 5	87 ± 15
**13b**	Br	H	-	<25	7.53 ± 0.97	>50	>6.6	61 ± 19	90 ± 5
**13c**	H	F	-	>25	>20	n.d.	-		

* n.d. not determined. ^a^ Compounds with inhibition at concentrations higher than 25 µM were considered not very active and were discarded for subsequent assays.

**Table 6:** Biological activity of quinolino[4,3-*b*][1,8] naphthyridines. The antileishmanial effect was evaluated in axenic IRFP-*L.infantum* amastigotes from bone marrow cells and subsequently in intramacrophagic IRFP-*L.infantum* from murine spleens. Cytotoxicity was assessed in uninfected splenic macrophages and intestinal tolerance in murine gut organoids.

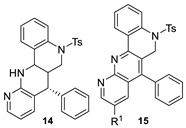							
Compound	R^1^	Axenic Amastigotes ^a^	Intracelullar Amastigotes EC_50_ (µM)	Mouse Splenic Macrophages CC_50_ (µM)	SI	Intestinal Organoids	
						50 µM (%Viability)	25 µM (%Viability)
**14a**	H	<25	9.34 ± 0.77	27.19 ± 1.55	2.9	56 ± 14	90 ± 9
**15a**	H	<25	67.43 ± 9.78	8.78 ± 2.21	0.1		
**15b**	Br	<25	6.16 ± 0.54	24.64 ± 3.59	4.0	13 ± 3	58 ± 16

^a^ Compounds with inhibition at concentrations higher than 25 µM were considered not very active and were discarded for subsequent assays.

## Data Availability

Data is contained within the article and Supplementary Materials.

## Supplementary Materials

The following supporting information can be downloaded at: https://www.mdpi.com/article/10.3390/molecules29010074/s1, Figure S1: Relaxation of supercoiled plasmid DNA assay.
