# Supplementary material for: Antileishmanial Effect of 1,5- and 1,8-Substituted Fused Naphthyridines
Source: Molecules. 2023 Dec 22;29(1):74. doi: 10.3390/molecules29010074 (PMC10780244; doi:10.3390/molecules29010074)
Supplement: Supplementary file 1 [file molecules-29-00074-s001.zip › molecules-2728604-supplementary.pdf]

Supplementary Material

Antileishmanial Effect of 1,5- and 1,8-Substituted Fused Naphthyridines

Estela Melcón-Fernandez [1], Endika Martín-Encinas [2], Francisco Palacios [2], Gulio Galli [1], Rosa M. Reguera [1], María Martínez-Valladares [1], Rafael Balaña-Fouce [1], Concepción Alonso [2,*] and Yolanda Pérez-Pertejo [1,*]

[1] Departamento de Ciencias Biomédicas, Facultad de Veterinaria, Universidad de León, Campus de Vegazana s/n, 24071 León, Spain; ggal@unileon.es (G.G.)

[2] Departamento de Química Orgánica I, Facultad de Farmacia, Lascaray Research Center, Universidad del País Vasco/Euskal Herriko Unibertsitatea (UPV/EHU), Paseo de la Universidad 7, 01006 Vitoria-Gasteiz, Spain

* Correspondence: concepcion.alonso@ehu.eus (C.A.); myperp@unileon.es (Y.P.-P.)

Relaxation of supercoiled plasmid DNA assay page 2

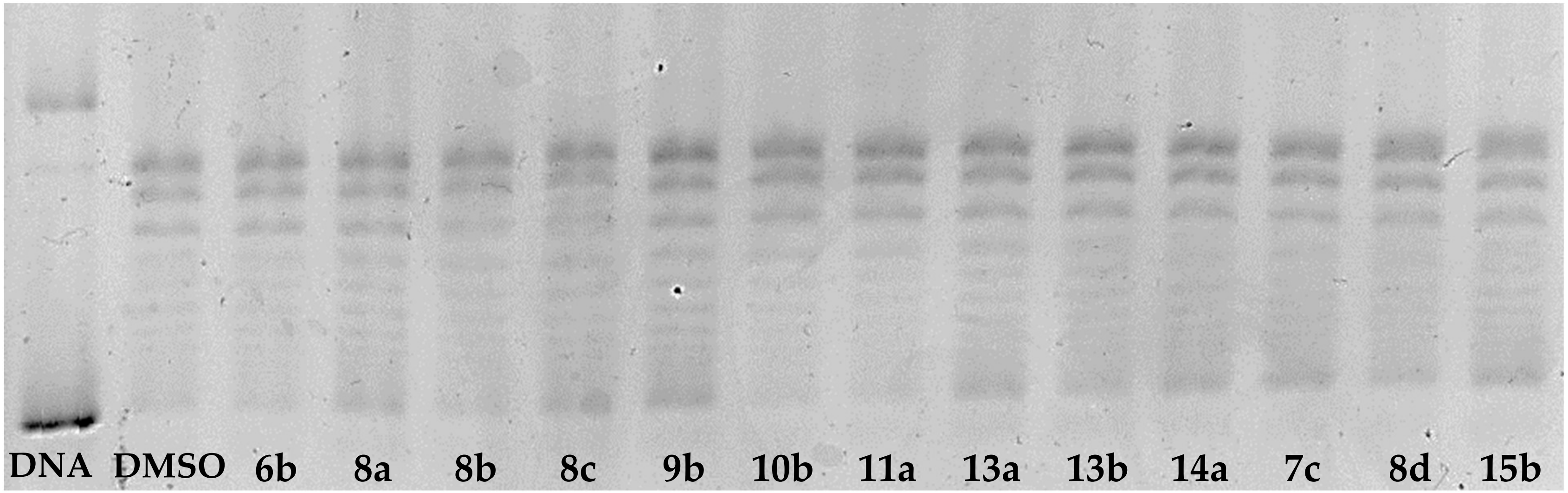

Relaxation of supercoiled plasmid DNA assay.

Each reaction contains one unit of purified leishmanial DNA topisomerase IB enzyme (LdTopIB), 100 μM of compounds, 0.5 μg of supercoiled pSK DNA, 10 mM Tris-HCl buffer pH 7.5, 5 mM $MgCl_2$, 0.1 mM EDTA, 15 μg/mL bovine serum albumin, 150 mM KCl. Reaction mixtures were incubated 1 min at 37 ºC and stopped by the addition of 4 μL loading buffer (5% sarkosyl, 0.12% bromophenol blue, 25% glycerol). The topoisomers were resolved in 1% agarose gels by electrophoresis in 0.1 M Tris borate EDTA buffer (pH 8.0) at 2 V/cm for 16 h and visualized with UV illumination after ethidium bromide (0.5 μg/mL) staining.
